# Acupuncture Reversible Effects on Altered Default Mode Network of Chronic Migraine Accompanied with Clinical Symptom Relief

**DOI:** 10.1155/2019/5047463

**Published:** 2019-03-18

**Authors:** Yan Zou, Weijun Tang, Xiang Li, Manwen Xu, Ji Li

**Affiliations:** ^1^Department of Integrated Traditional and Western Medicine, Huashan Hospital, Fudan University, Shanghai, China; ^2^Department of Radiology, Huashan Hospital, Fudan University, Shanghai, China; ^3^Department of Neurology, Huashan Hospital, Fudan University, Shanghai, China

## Abstract

**Objective:**

To determine whether and how longitudinal acupuncture modulates the impaired default mode network (DMN) in chronic migraine (CM) patients without aura.

**Methods:**

Resting-state functional magnetic resonance imaging (fMRI) data from 14 CM patients treated with longitudinal pre- and postacupuncture treatment (PPAT) and data of 18 age- and gender-matched healthy controls (HCs) were analyzed using independent component analysis (ICA) and seed-based correlation analysis (SCA) to investigate connectivity within the DMN. Correlation analyses were performed to identify associations between changes in functional connectivity (FC) and in clinical pain based on PPAT observations. The monthly mean visual analog scale (VAS) scores, monthly mean headache attacks, monthly headache days, monthly amount of acute headache medications, and immediate VAS scores were assessed for evaluation of pain.

**Results:**

The decreased FC within the DMN found in the left superior prefrontal gyrus (L_SPFG) and left precuneus (L_PRECUN) of CM patients was returned to the healthy control level after acupuncture treatments. Furthermore, the diminished pairwise FC strengths in some regions of interest (ROIs) within the DMN were also increased, mainly distributed between the right temporal lobe (R_TPL) and left anterior cingulate cortex, between the R_TPL and bilateral superior medial gyrus, and between the R_TPL and right precuneus. Increased *z*-scores within the DMN (L_SPFG and L_PRECUN) were associated with reduced immediate VAS scores, and increases in *z*-scores of the L_PRECUN were negatively correlated with reductions in the monthly amount of acute headache medications. However, no association existed between the increased DMN connectivity and reduced monthly mean VAS scores, monthly mean headache attacks, and monthly headache days.

**Conclusion:**

Altered DMN connectivity and its normalization postacupuncture can be employed to monitor CM and its modulating effects. The DMN is useful for understanding the therapeutic mechanisms of acupuncture in CM.

## 1. Introduction

Chronic migraine (CM) is a common disabling neurological disorder characterized by recurrent headache accompanied with sensory, emotional, and cognitive symptomology [1]. The global prevalence of CM ranges from 1.4% to 2.2%, and approximately 2.5% of episodic migraine (EM) cases transform to CM every year, resulting in an enormous economic burden on individuals and society [2, 3]. At present, many adverse events (abuse, addiction, and dependence) have been reported with the long-term use of conventional treatments for CM such as prophylactic or analgesic drugs [4, 5]. Alternatively, acupuncture has gained popularity for its pronounced and long-lasting therapeutic effect against CM. Evidence from clinical studies indicates that acupuncture can be at least as effective as prophylactic drugs for migraine and elicits with very few side effects [6–9]. However, because the progression of migraine into a chronic condition is poorly understood, the therapeutic mechanism of acupuncture for CM remains elusive and restricts its further popularization and application. Thus, an improved understanding of CM will open a new avenue for elucidating the mechanism of acupuncture treatment.

The default mode network (DMN) is a constellation of brain regions and engages in higher-order cognitive functions including self-referential processing, episodic memory retrieval, and internal and external environment monitoring [10–12]. It is more active during rest than upon exposure to external stimuli [13, 14]. Recent resting-state functional magnetic resonance imaging (rs-fMRI) studies on the brains of patients with different chronic pain disorders revealed drastic changes in the DMN properties, which predict that the dynamics of the DMN are maladaptive in patients with chronic pain [15, 16]. To date, studies have used different analytical methodologies to assess the functional connectivity (FC) in migraine. Using independent component analysis (ICA), Tessitore et al. reported diminished connectivity in the prefrontal cortex and temporal cortex within the DMN in migraine patients without aura [17], while Xue et al. noted that migraine was associated with more intense connectivity between the DMN, central executive network (CEN), and insular cortex [18]. Another study on migraine using regional homogeneity (ReHo) analysis also demonstrated that core regions of the DMN (e.g., anterior and posterior cingulate cortex) were deactivated [19]. These conflicting findings may have resulted from the use of different parameters or approaches. It is noteworthy that very few of these studies specifically focused on the integrity of DMN connectivity in CM patients, and none investigated the DMN dynamics and how DMN connectivity correlates with relief from clinical pain throughout the entire treatment procedure.

Therefore, in the present study, we chose dynamic changes in the DMN as a biomarker for the effectiveness of acupuncture treatment. We hypothesized that (1) CM is associated with dysfunction of the DMN and the impaired DMN connectivity can be normalized after longitudinal treatments and (2) changes in connectivity within the DMN correlate with alterations in the clinical data (pain intensity, frequency of headache attacks, headache days, and amount of acute headache medications) of CM patients.

## 2. Methods

### 2.1. Patient Recruitment

CM patients without aura (9 female and 5 male patients, aged 42.91 ± 10.18 years) were recruited over 3 years in the Department of Neurology and Traditional Chinese Medicine (TCM) of Huashan Hospital, Fudan University. Healthy controls (HCs, 9 female and 9 male participants, aged 38.59 ± 7.96 years) were either hospital staff or volunteers from the community. All participants provided informed written consent according to our protocol that was approved by the Institutional Review Board at Huashan Hospital of Fudan University.

### 2.2. Inclusion Criteria

The inclusion criteria included the following: (1) CM without aura that fulfilled the International Classification of Headache Disorders III (ICHD-3 beta) [1]: headache occurring with a frequency of 15 or more days per month for >3 months, of which at least 8 days had the features of migraine headache; (2) an available, patient-recorded daily diary of headache (including frequency of headache attacks, headache days, amount of acute headache medications, and pain intensity); (3) clinical pain assessment with a VAS score > 4 (range, 0–10); and (4) no acupuncture treatment during the preceding month.

### 2.3. Exclusion Criteria

The exclusion criteria included the following: (1) participation in another clinical study before acupuncture treatment or overuse of pain medication; (2) any other neurological or psychiatric disorder; (3) pregnancy or lactation; (4) other types of headache; (5) history of head trauma or brain tumor; (6) contraindication for MRI scanning; and (7) use of any preventive medications for migraine.

### 2.4. Acupuncture Treatment

Eight acupoints were selected for the standardized acupuncture protocol: *SJ5* (*bilateral*), *GB20* (*bilateral*), *GB8* (*bilateral*), and *ST8* (*bilateral*). Disposable stainless-steel acupuncture needles (0.22 × 25 mm) were inserted into the principal acupoints, to a depth of 5–15 mm to elicit the *de qi* sensation. Each stimulation lasted for 30 minutes. Patients received 36 acupuncture treatments thrice weekly for 3 months.

### 2.5. Assessment of Clinical Pain

Monthly mean VAS scores, monthly mean headache attacks, monthly headache days, monthly amount of acute headache medications, and immediate VAS scores (before each fMRI scan) were assessed.

### 2.6. fMRI Examination

CM patients received two fMRI scans (during migraine before treatment and on the second day at the end of all treatments). Healthy controls received one fMRI scan.

### 2.7. Acquisition of Imaging Data

fMRI images were acquired using a GE Signa VH/i 3.0T scanner. Participants were instructed to keep their eyes closed and avoid falling asleep. High-resolution T1-weighted anatomical images were acquired using a fast spoil gradient recall sequence: TR = 2300 ms, TE = 2.98 ms, flip angle = 9°, FOV = 256, matrix = 256 × 256, and slice thickness = 1 mm. Functional images were acquired using a T2∗-weighted echo planar image (EPI) sequence: TR = 2000 ms, TE = 35 ms, flip angle = 90°, matrix = 64 × 64, FOV = 256, slice thickness = 4 mm, gap = 0 mm, and 200 time points.

#### 2.7.1. Preprocessing of Imaging Data

Data were preprocessed using Data Processing & Analysis for Brain Imaging (DPABI, http://rfmri.org/dpabi) toolbox. The preprocessing steps included the following: (1) removal of the first 10 time points; (2) slice-timing correction; (3) head motion realignment; (4) coregistration of each participant's functional and anatomical images; (5) spatial normalization to Montreal Neurological Institute (MNI) space using diffeomorphic anatomical registration through exponentiated lie algebra (DARTEL) tool with a voxel size of resampled images of 3 mm × 3 mm × 3 mm; and (6) smoothing of all functional images with 6 mm FWHM Gaussian kernel.

#### 2.7.2. Postprocessing of Imaging Data

Spatial independent component analysis (ICA) was conducted for all preprocessing data using the GIFT software (Group ICA Toolbox, http://mialab.mrn.org/software/gift/), comprising mainly (1) independent component (IC) estimation with minimum description length (MDL) criteria [20], (2) ICA separation using the infomax algorithm, (3) back reconstruction of corresponding individual participating components and time courses from aggregate components, (4) DMN spatial component selection from all ICs, and (5) transformation of the intensity values in each IC spatial map to *z*-scores.

Seed-based correlation analysis (SCA) was also performed. After the preprocessing steps, the DPABI toolbox was used to fit the data to a linear trend and apply band-pass filtering (0.01–0.08 Hz) to extract low-frequency fluctuations. To reduce interference from physical noise, such as head motion, respiration, and cardiac rhythms, multiple linear regression analyses were used to remove several nuisance signals, including six head motion parameters, white matter signal, and cerebrospinal fluid signal. Bilateral anterior cingulate cortex (ACC), bilateral precuneus (PRECUN), bilateral superior medial gyrus (SMG), bilateral superior prefrontal gyrus (SPFG), and bilateral temporal lobe (TPL) were selected as regions of interest (ROIs) for SCA (Table 1). All ROIs were core nodes of the separated DMN spatial map and were obtained by creating spheres with a 10 mm diameter. The signal time series of all voxels within each ROI were extracted. The pairwise correlation coefficient (*r* value) between ROIs was calculated for each participant to compare changes in FC. All FC images were converted into *z*-scores by Fisher's *Z* transformation.

### 2.8. Statistical Analysis

#### 2.8.1. Analysis of Imaging Data

For ICA analysis, one-sample *t*-tests were performed to determine DMN spatial maps among all the groups (HC, Pre-, and Posttreatment groups). Independent-sample *t*-tests were performed to compare intergroup differences within DMN maps between the HC and Pretreatment groups as well as between the HC and Posttreatment groups. Paired-sample *t*-tests were performed to compare intragroup differences within DMN maps between the Pre- and Posttreatment groups. The threshold for statistical significance was set at *P* < 0.05 (AlphaSim correction).

For SCA analysis, independent-sample *t*-tests were performed to detect pairwise changes in FC between the HC and Pretreatment groups as well as between the HC and Posttreatment groups. Paired-sample *t*-tests were also performed to compare changes in FC between the Pre- and Posttreatment groups. The threshold for statistical significance was set at *P* < 0.05 (network-based connection correction).

#### 2.8.2. Analysis of Clinical Data

The mean DMN *z*-scores (component time course-related activities) of each group for persisting clusters of CM (Pre- and Posttreatment and HC groups) were extracted for correlation analyses with monthly mean headache attacks, monthly mean VAS scores, monthly headache days, monthly amount of acute headache medications, and immediate VAS scores. In addition, the changes in monthly mean headache attacks, monthly mean VAS scores, monthly headache days, or monthly amount of acute headache medications between the Pre- and Posttreatment groups were analyzed.

## 3. Results

### 3.1. Clinical Information for All Participants

**Figure 1:**
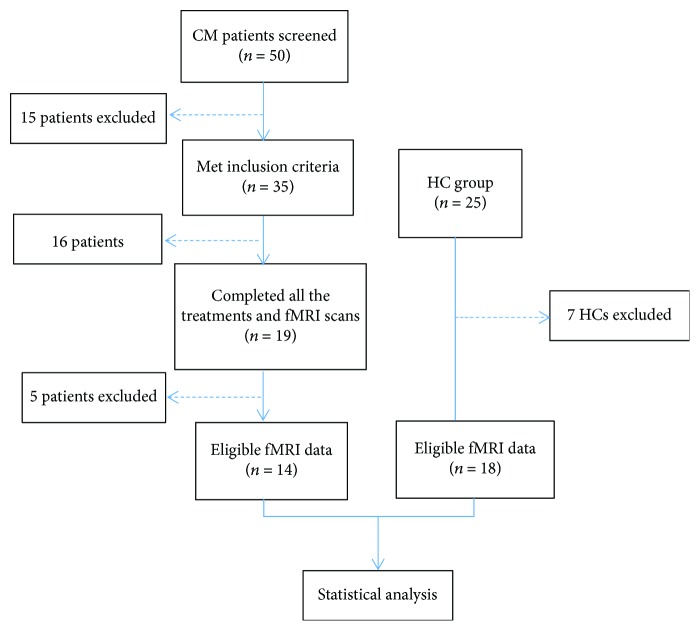
HC: healthy controls; CM: chronic migraine; fMRI: functional magnetic resonance image; *n*: number.

As illustrated in Figure 1, at the very beginning, 50 CM patients were screened and only 35 met the requirements. Then, 16 patients were excluded during the acupuncture treatment period for various reasons (e.g., failure to complete all treatments and use of other medications). Five CM patients and seven HCs were excluded for ineligible fMRI data. Finally, only the data of 14 CM patients and 18 HCs could be used for analysis. The clinical information of the final participants is shown in Table 2. There were no obvious differences in age and gender (*P* > 0.05), and there were statistically significant reductions from before to after treatment in the monthly mean headache attacks, monthly mean VAS scores, monthly headache days, monthly amount of acute headache medications, and immediate VAS scores (*P* < 0.01).

### 3.2. Altered DMN Integrity in the CM Group Compared with the HC Group

**Figure 2:**
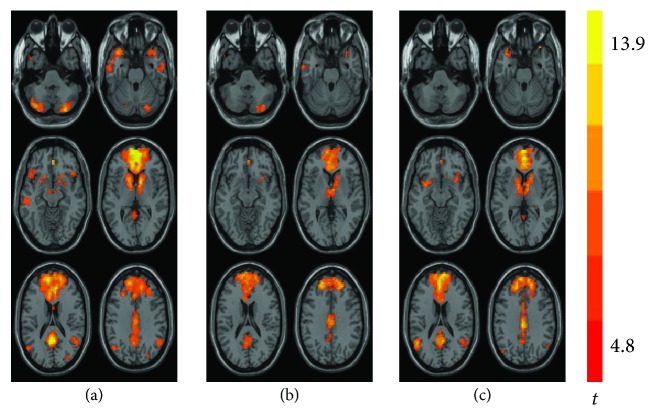
Group level of DMN connectivity observed in the HC group (a), Pretreatment group (b), and Posttreatment group (c). *P* < 0.05, AlphaSim corrected. DMN: default mode network; HC: healthy control; CM: chronic migraine.

**Figure 3:**
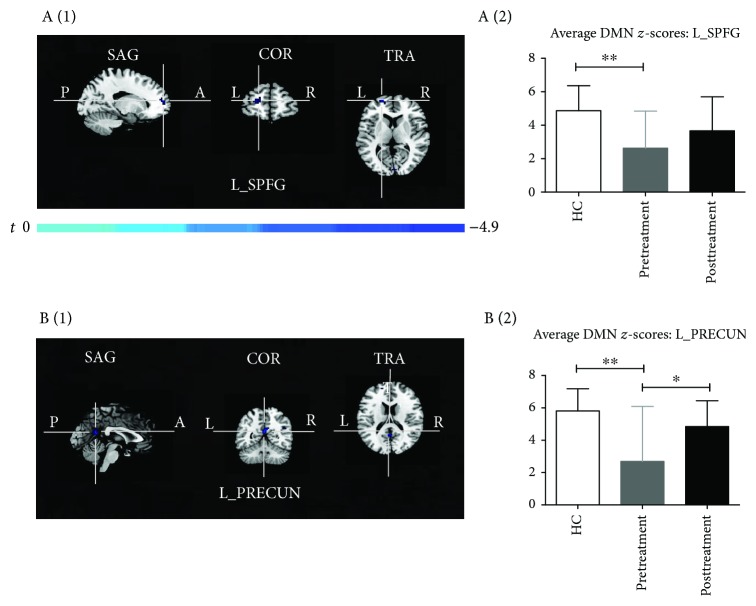
(a (1), b (1)) T-maps of differences in DMN connectivity between the Pretreatment group and HC group (*P* < 0.05, AlphaSim corrected). (a (2), b (2)) Bar graphs of the mean DMN *z*-scores of the group's different regions (L_SPFG and L_PRECUN). DMN: default mode network; CM: chronic migraine; HC: healthy controls; L_SPFG: left superior prefrontal gyrus, MNI coordinates (*x*, *y*, and *z*): -21, 60, and 9, respectively; L_PRECUN: left precuneus, MNI coordinates (*x*, *y*, and *z*): 0, -54, and 18, respectively; ^∗^*P* < 0.05 and ^∗∗^*P* < 0.01.

As illustrated in Figure 2, a DMN map was obtained for each group (HC, Pre- and Posttreatment groups). In the HC group, the observed increase in the scope of DMN connectivity was consistent with prior reports, encompassing the PRECUN, ACC, TPL, SPFG, SMG, and cerebellar areas (Figure 2(a)). Comparison of the DMN connectivity in the Pretreatment group versus the HC group revealed significant reductions in the left SPFG (L_SPFG, MNI coordinates *x*, y, and *z*: -21, 60, and 9, respectively) and left PRECUN (L_PRECUN, MNI coordinates *x*, *y*, and *z*: 0, -54, and 18, respectively), accompanied by decreased DMN *z*-scores within the two different regions (Figures 2(b) and 3). In addition, in the Posttreatment group, the lessened connectivities within the DMN and the corresponding mean DMN *z*-scores were almost reversed and there were no significant differences between the HC and Posttreatment groups (Figures 2(c) and 3).

### 3.3. Abnormal Pairwise FC of Selected ROIs in the CM Group Compared with the HC Group

**Figure 4:**
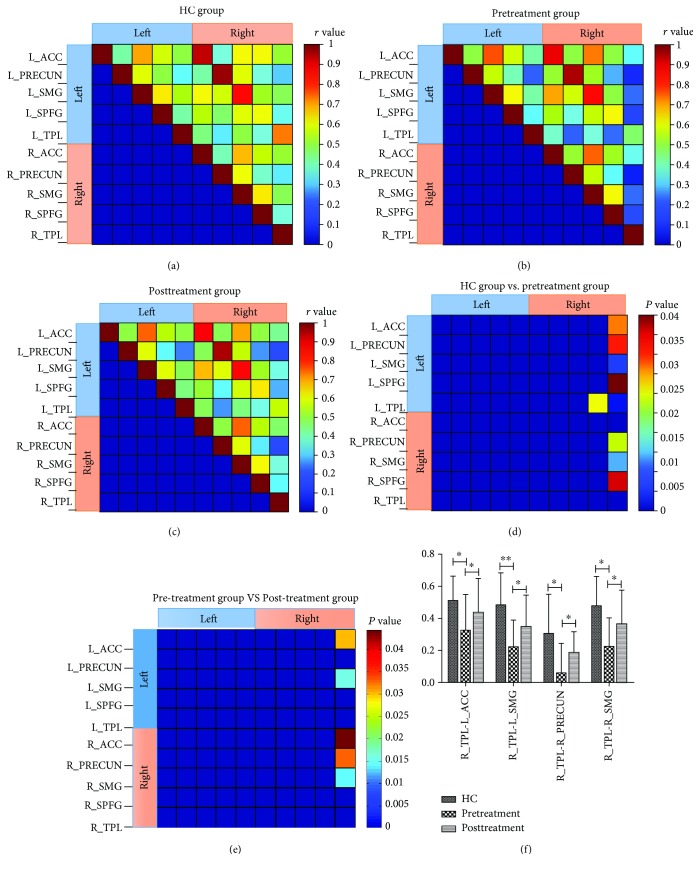
Matrix plot of mean pairwise FC of 10 ROIs from HC (a), Pretreatment (b), and Posttreatment (c) groups. Pairwise FC differences between the HC group and Pretreatment group (d) and between the Pretreatment group and Posttreatment group (e). Bar graphs of pairwise FC changes (*r* value) of altered ROIs in the HC, Pretreatment, and Posttreatment groups (f). ^∗^*P* < 0.05 and ^∗∗^*P* < 0.01.

As illustrated in Figures 4(a)–4(c), a matrix plot of mean pairwise FC in 10 ROIs was prepared for each group (the HC, Pre-, and Posttreatment groups). Compared with the HC group, the Pretreatment group exhibited a decreased FC (1) between the R_TPL and L_ACC, between the R_TPL and bilateral PRECUN, between the R_TPL and bilateral superior medial gyrus (SMG), between the R_TPL and bilateral SPFG, and (2) between the R_SPFG and L_TPL (Figure 4(d)). After longitudinal acupuncture treatments, there was an obvious increase in the attenuated pairwise FC of some ROIs, mainly distributed between the R_TPL and L_ACC, between the R_TPL and bilateral SMG, and between the R_TPL and R_PRECUN (Figures 4(e) and 4(f)).

### 3.4. Correlation between *z*-Scores of Altered Regions

**Figure 5:**
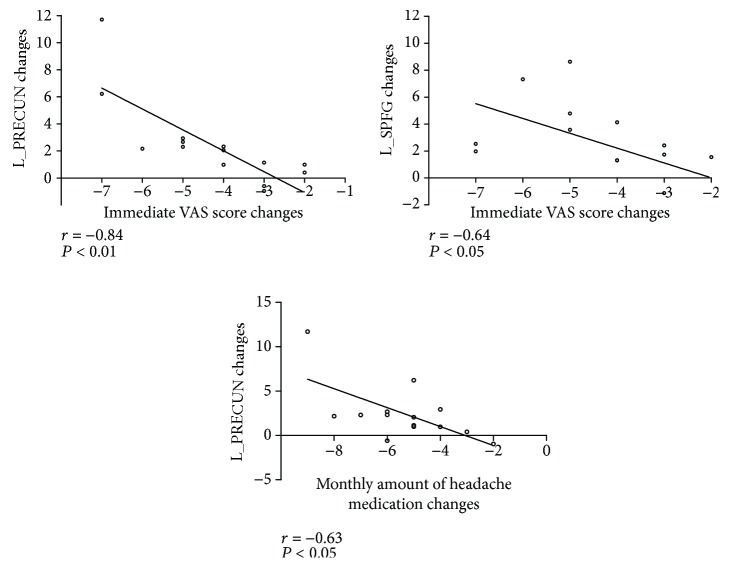
Scatter graphs of changes in mean DMN *z*-scores for different regions (L_SPFG and L_PRECUN) and changes in immediate VAS scores and monthly acute headache medications of patients. The changes of *z*-scores of the L_SPFG and L_PRECUN were negatively correlated with reductions in immediate VAS scores. In addition, the increases in *z*-scores of L_PRECUN were also negatively correlated with the reductions in the monthly amount of acute headache medications used. The changes between *z*-scores of the L_SPFG and immediate VAS scores of 3 patients were the same.

**Figure 6:**
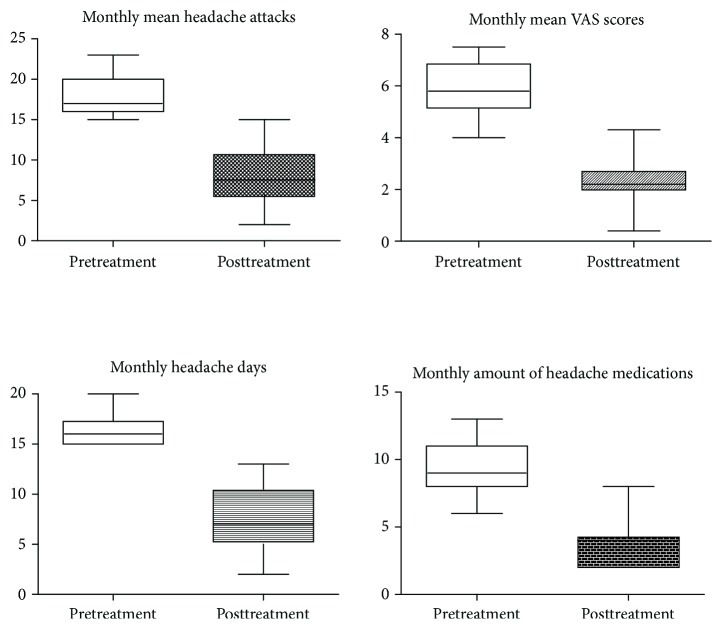
The differences in the monthly mean headache attacks, monthly mean VAS scores, monthly headache days, and monthly amount of acute headache medications between the Pretreatment and Posttreatment groups were statistically significant and obvious, exhibiting a decrease after longitudinal treatment.

The correlation between the changes in the mean DMN *z*-scores within the decreased regions (L_SPFG and L_PRECUN) and the alterations in monthly mean VAS scores, monthly mean headache attacks, and monthly headache days were not statistically significant, but these functional alterations were negatively correlated with the change in immediate VAS scores and monthly amount of acute headache medications (Table 3 and Figure 5). The differences in the monthly mean headache attacks, monthly mean VAS scores, monthly headache days, and monthly amount of acute headache medications between the Pre- and Posttreatment groups were significant and obvious, showing a decline after longitudinal acupuncture treatments (Figure 6).

## 4. Discussion

This is the first study combining ICA and SCA to explore the changes in the whole DMN integrity and pairwise FC of core nodes within the DMN. In addition, correlation analyses were performed to detect relationships between changes in DMN connectivity and alterations in clinical data (including monthly mean VAS scores, immediate VAS scores, monthly mean headache attacks, monthly headache days, and monthly amount of acute headache medications).

In the comparison of Pre- and Posttreatment fMRI data, we identified two core regions of the DMN, namely, the L_SPFG and L_PRECUN, showing reduced FC in patients with CM, and these areas could be almost normalized to the HC level via longitudinal acupuncture treatments. These two areas are relevant for higher-order cognitive processes: the SPFG is critical for mediation of pain perception, whereas the PRECUN is an intriguing cortical area that may support a variety of behavioral functions such as episodic memory retrieval and self-referential goal-directed actions and is engaged in the regulation of pain affection or empathy [21–24]. Furthermore, for a better understanding of the interregional FC within the DMN, 10 regions were selected based on the separated DMN of HC as ROIs to detect differences among the three groups. We found that after longitudinal acupuncture treatments, decreased pairwise FC of some ROIs was conspicuously increased, mainly distributed between the R_TPL and L_ACC, between the R_TPL and bilateral SMG, and between the R_TPL and R_PRECUN. The TPL plays an important role in episodic memory and pain perception. The increased pairwise FC between the TPL and other cognitive regions (included ACC, SMG, and PRECUN) could also reflect the modulating effect of acupuncture on CM patients, which is consistent with the changes in DMN integrity.

Mounting evidence indicates that pain may be detrimental to the brain and that long-term pain itself can both impair an individual's ability to control the pain and alter cognitive and emotional modulation of pain, leading to a higher pain intensity and/or a longer pain duration [25–27]. Conversely, a positive emotion can have a notable impact on the perception of pain [25, 28]. We presume that the cumulative effects of multiple acupuncture treatments will gradually modulate the cognitive and affective aspects of pain, which may help to reverse brain changes associated with chronic pain persistence.

A comparison of Pre- and Posttreatment clinical data demonstrated that the treatment brought about significant reductions in the monthly headache attacks, monthly mean VAS scores, monthly headache days, and monthly amount of acute headache medications, reflecting the clinical relief of CM patients due to the acupuncture treatment. Moreover, our results (both L_PRECUNE and L_SPFG) indicated that the lower immediate VAS scores correlated with the enhanced DMN connectivity, which was similar to our previous findings [29]. The reductions in the monthly amount of acute headache medications were also negatively correlated with increases of L_PRECUN connectivity. However, these functional changes within the DMN and changes in the monthly mean VAS scores, monthly mean headache attacks, or monthly headache days did not reveal any obvious association. Immediate VAS scores were measured before each scan and could reflect changes in the instant DMN connectivity. Monthly mean headache attacks, monthly mean VAS scores, monthly headache days, and monthly amount of acute headache medications reflected monthly dynamic changes in the pain level. Although there were negative correlations between changes in the monthly amount of acute headache medications and DMN connectivity, other clinical indicators did not reveal any correlation with changes of DMN connectivity. Hence, the relationship between changes in DMN connectivity and dynamic changes in pain level requires further research.

The imaging and clinical results obtained in this study provide convincing evidence to characterize CM as a brain dysfunction affecting both DMN integrity and its internal FC pathway, likely reflecting the deleterious influence of constant pain on brain function. However, this study raises several issues. Firstly, the sample size was relatively small in each group, and the selection of acupoints was based on conventional clinical practice. Nevertheless, all patients' first fMRI scans were conducted during the period of headache attack, and all patients were subjected to long-term follow-up observations. In addition, this study was aimed at investigating the therapeutic effects of acupuncture on the disrupted DMN connectivity in CM patients rather than the effects of the compatibility of acupoints. Our study can provide potentially objective evidence from an investigation of CM and its modulating effects. Secondly, although use of the VAS is a simple method for testing for clinical pain, especially chronic pain, the effects of spontaneous pain on the accuracy of VAS scores during the time of the scan are unknown. Thirdly, we have not clearly confirmed whether the functional alterations were in line with structural alterations in the brain. Thus, a multimodal analysis that contains both structural data analysis and functional data analysis should be conducted in future studies.

## 5. Conclusion

We believe that shifting of the altered DMN connectivity to its renormalization via intervention can be used to track chronic pain and its modulating effect. Furthermore, DMN connectivity is conducive to an understanding of the brain correlates of the therapeutic effects on CM. Thus, the clinical efficacy of acupuncture can be monitored and evaluated through DMN connectivity.

## Figures and Tables

**Table 1:** ROIs within the separated DMN components.

Name of the region	Abbreviation	Extent	MNI coordinates		
			*x*	*y*	*z*
Left anterior cingulate cortex	L_ACC	171	-6	39	3
Left precuneus	L_PRECUN	168	-6	-54	18
Left superior medial gyrus	L_SMG	171	-9	54	6
Left superior prefrontal gyrus	L_SPFG	121	-21	60	9
Left temporal lobe	L_TPL	101	-39	18	-27
Right anterior cingulate cortex	R_ACC	171	6	39	3
Right precuneus	R_PRECUN	168	6	-54	18
Right superior medial gyrus	R_SMG	171	9	54	6
Right superior prefrontal gyrus	R_SPFG	140	-21	60	9
Right temporal lobe	R_TPL	98	-39	18	-27

**Table 2:** Clinical details of the HC and CM groups.

	HC group	CM group	
	—	Pretreatment	Posttreatment
Number of cases, *n*	18	14	
Male, *n*	9	5	
Female, *n*	9	9	
Age (years), mean ± SD	38.59 ± 7.96	42.91 ± 10.18	
VAS score (monthly), mean±SD^∗∗^	—	5.91 ± 0.98	2.28 ± 0.97
Headache attacks (monthly), mean±SD^∗∗^	—	17.42 ± 2.27	7.53 ± 3.38
Immediate VAS score, mean±SD^∗∗^	—	5.38 ± 1.19	1 ± 0.91
Headache days (monthly), mean±SD^∗∗^	—	16.36 ± 1.60	7.50 ± 3.37
Acute headache medications (monthly), mean±SD^∗∗^	—	9.29 ± 1.98	3.93 ± 1.82

Results represent mean ± standard deviation; ^∗∗^*P* < 0.01.

**Table 3:** Correlations of changes in L_PRECUN and L_SPFG *z*-scores and changes in clinical data.

	L_PRECUN *z*-score changes	L_SPFG *z*-score changes
Monthly mean VAS score change, mean ± SD	*r* = −0.172, *P* = 0.557	*r* = 0.224, *P* = 0.441
Monthly headache attack changes, mean ± SD	*r* = −0.302, *P* = 0.293	*r* = 0.069, *P* = 0.814
Monthly headache day changes, mean ± SD	*r* = −0.002, *P* = 0.993	*r* = 0.017, *P* = 0.955
Monthly amount of acute headache medication changes, mean ± SD	*r* = −0.626, *P* = 0.017^∗^	*r* = −0.499, *P* = 0.070
Immediate VAS score changes, mean ± SD	*r* = −0.839, *P* = 0.0002^∗∗^	*r* = −0.641, *P* = 0.013^∗^

*r* = correlation coefficient; results represent mean ± standard deviation; ^∗∗^statistically significant difference, *P* < 0.01; ^∗^statistically significant difference, *P* < 0.05.

## Data Availability

The datasets analyzed during the current study are not publicly available due to the unfinished study of the whole project but are available from the corresponding author on reasonable request.

## Additional Points

*Provenance and Peer Review*. This study was not commissioned; it was externally peer reviewed.
